# Low infliximab serum trough levels and anti-infliximab antibodies are prevalent in rheumatoid arthritis patients treated with infliximab in daily clinical practice: results of an observational cohort study

**DOI:** 10.1186/1471-2474-13-184

**Published:** 2012-09-24

**Authors:** Aatke van der Maas, Bart JF van den Bemt, Gertjan Wolbink, Frank HJ van den Hoogen, Piet LCM van Riel, Alfons A den Broeder

**Affiliations:** 1 Department of Rheumatology, Sint Maartenskliniek, Hengstdal 3, 6522 JV, Nijmegen, The Netherlands; 2 Department of Pharmacy, Sint Maartenskliniek, Hengstdal 3, 6522 JV, Nijmegen, The Netherlands; 3 Department of Rheumatology, Reade, Dr. Jan van Breemenstraat 2, 1056 AB, Amsterdam, The Netherlands; 4 Department of Rheumatology, Radboud University Nijmegen Medical Center Geert Grooteplein-Zuid 10, Postbus 9101, 6500 HB, Nijmegen, The Netherlands

**Keywords:** Rheumatoid arthritis, Infliximab, Therapeutic drug monitoring, Serum trough levels, Anti-infliximab antibodies, Low disease activity

## Abstract

**Background:**

To get insight in the prevalence of high, or low/no serum infliximab trough levels in patients with low disease activity and if serum trough levels are stable and reliable longitudinally we conducted a prospective cohort study

**Methods:**

In a longitudinal, observational cohort of RA patients treated with infliximab for at least 6 months, treatment interval, DAS28, infliximab trough levels and anti-infliximab antibodies were assessed. Prevalence of low (<1 mg/l) and high (>5 mg/l) infliximab serum trough levels and anti-infliximab antibodies was recorded. Relationship of a change in anti-infliximab antibodies and treatment interval was described. Reliability of consecutive infliximab serum trough levels and anti-infliximab antibodies in patients with stable DAS28 and treatment was analysed with Spearman correlation and kappa-analysis.

**Results:**

147 patients with a mean disease duration of 11 years (sd7) and DAS28 of 3.5 (sd1.3) at baseline were followed during 1.5 years. Inter-individual variability in infliximab levels in patients with low DAS28 was high (median 1.4 mg/L, IQR 3.35), with 31% (95%CI: 20-42%) having low (<1 mg/L) and 14% (95%CI 5–22) high trough levels (>5 mg/L). Interestingly also in RA patients with DAS28 ≤ 3.2, anti-infliximab antibodies were found in one-third of the patients, with half of them having antibodies every visit during a median of more than one year. Agreement for consecutive measurements of serum trough levels and anti-infliximab antibodies was high in stable patients: r = 0.97 (p = 0.00001) and kappa = 1.0 (SE 0.14) Anti-infliximab antibody appearance was influenced by interval increases (relative risk (RR) 5.2, 2.6-10.7), but patients still showed low infliximab levels.

**Conclusions:**

Low (and high) infliximab serum trough levels are prevalent, interestingly also in patients with low disease activity. Consecutive measurements of serum trough levels and anti-infliximab antibodies are reliable in stable patients. These test could be used to lower or stop infliximab in selected patients.

## Background

Infliximab, a chimeric (human-mouse) monoclonal antibody to human tumour necrosis factor-α (TNF-α), has proved to be effective in the treatment of rheumatoid arthritis (RA) in several pivotal randomised controlled trials [1,2]. There is however a difference in response between individual RA patients, both in the initiation and in the maintenance phase. Differences in response to infliximab in the initiation phase could be partly explained by inter-individual differences in pharmacokinetics as reflected in lower infliximab trough levels and presence of anti-infliximab antibodies in patients not responding to infliximab [3-7].

In the maintenance phase of treatment with infliximab there are still large inter-individual differences in disease activity, as witnessed by the relatively high mean and large variation in disease activity scores (mean DAS28 in the DREAM registry after one year was about 4) and large proportion of patients with high disease activity in biological registries (for example about 45% in the NOR-DMARD database had a DAS28 > 3.2 after 6 months) [8,9]. This can be explained by inadequate initial response, but also by the occurrence of secondary loss of response after initial improvement on infliximab. Therefore further improvement of treatment regimens seems warranted.

The first optimisation would of course be to switch patients not doing well to another biological. Another form of treatment optimisation, however, could be lowering the dose or stopping infliximab in patients in whom infliximab is either given in a too high dose, or in whom the drug is no longer effective. Indeed, stop or dose reduction studies have shown that this is feasible in a large proportion of patients [10-12]. It would be very helpful when successful dose reduction or stopping could be predicted in these patients, to prevent unnecessary flares. Such a predictor is however not yet available.

Recently several studies also demonstrated the potential use of monitoring of pharmacokinetics during the maintenance phase of infliximab treatment in RA patients, next to the initiation phase [13-16]. Therefore, an interesting possible predictor for successful dose tapering could be infliximab serum trough levels and anti-infliximab antibody levels. It can be conceived that patients with very high serum trough levels of infliximab could be carefully dose reduced, and that on the other hand patients without detectable infliximab trough levels (or anti-infliximab antibodies) could even stop the drug without deterioration of disease activity. However, there is not much data on the potential value of measuring serum trough levels and anti-infliximab antibodies to guide infliximab treatment in daily clinical practice during the maintenance phase. Therefore, to get insight in the prevalence and course of infliximab serum trough levels and anti-infliximab antibodies in patients with low disease activity, we conducted a prospective observational longitudinal cohort study focussing on two research questions:1) what is the prevalence of high, or low/no serum infliximab trough levels in patients with low disease activity? 2) Are these serum trough levels stable and reliable longitudinally, also in the context of treatment interval changes?

## Methods

### Patients and measurements

All RA patients treated for at least 6 months (maintenance phase) with infliximab in daily clinical practice at the Sint Maartenskliniek, Nijmegen, The Netherlands were included in a longitudinal observational cohort during a 1.5 year period or until treatment was discontinued. Enrolment of patients in this dynamic cohort started in February 2007. All patients received 3 mg/kg, with infusion intervals varying from 4–12 weeks. Decisions on interval variation were made by the treating rheumatologist who was blinded for the results of the serum trough levels and anti-infliximab antibodies, but according to local treatment protocol a DAS28 was measured every visit and rheumatologists were advised to shorten the interval or change therapy if the DAS28 was more than 3.2 during 2 visits or lengthen the interval if the DAS28 was less than 2.6 during at least half a year. According to local protocol intervals were allowed to vary between 4–12 weeks. At every visit immediately before infusion sera were collected. Serum trough levels and anti-infliximab antibodies were measured as described elsewhere in one batch after the study had ended [6,7]. Low levels were defined as <1.0 mg/L and high levels as >5.0 mg/L. Both cut-offs are in accordance with previous reported thresholds [6].

Approval from the Medical Research Ethics Committee (MREC) was sought for. The committee decided that this approval was not necessary because DAS28 guided dose adaptation was performed as usual care for all patients meeting the requirements of the Dutch legislation and no extra venous puncture was necessary. Informed consent for the extra blood sample and use of medical and demographic data was obtained from all patients.

### Statistical analyses

Descriptive statistics were provided using mean (+/− standard deviation (SD)) or median (interquartile range(IQR)) values depending on the (non-) parametric distribution of measured patient characteristics. We explored our data by describing the following variables at study enrolment: prevalence of remission (defined as DAS28 ≤ 2.6), low (DAS28 ≤ 3.2) and high (DAS28 > 3.2) disease activity state, low (<1.0 mg/L) and high (>5.0 mg/L) infliximab serum trough levels and presence of anti-infliximab antibodies [6,7]. Also the percentage of visits with low disease activity and remission was described. Additionally, the prevalence of low serum trough levels and anti-infliximab antibodies was confirmed in a subgroup of patients with long duration of stable low disease activity, defined as a DAS28 ≤ 3.2 for at least 3 consecutive visits. Furthermore, the course of anti-infliximab antibodies during follow up was investigated longitudinally. The influence of an increase in interval on anti-infliximab antibody occurrence and the influence of a shorter interval on anti-infliximab antibody disappearance were analysed by means of relative risk ratios (RR). Concomitant methotrexate (MTX) use was taken into account in the analysis using relative risk ratios as well.

Finally, we also aimed to establish within-patient reliability of infliximab serum trough levels and anti-infliximab antibodies. To investigate this, infliximab serum trough levels measured at 2 consecutive visits in patients with stable DAS28 and stable treatment were compared with a Wilcoxon test and a spearman correlation test. Furthermore, mean difference and limits of agreement (LoA) were calculated [17]. The reliability of anti-infliximab antibodies was determined by kappa test statistic. All analyses were done with STATA 10.1 statistical software.

## Results

### Patient characteristics and infliximab serum trough levels

In total 1320 visits in 147 patients treated with infliximab for at least 6 months were collected in the observation period. Demographic data at study enrolment is depicted in Table 1. In 27% (95% confidence interval (CI): 20-34%) and 44% (95%CI:36-52%) of the patients the DAS28 was below 2.6 or 3.2 respectively. As demonstrated in Table 2, at study enrolment, low infliximab serum trough levels are prevalent in patients with DAS28 > 3.2 (48%, 95%CI: 37–59), but interestingly also in patients with low disease activity (31%, 95%CI: 20–42). In 14% of the patients with low DAS28 and in 21% of the patients with high DAS28 high infliximab serum trough levels were found (see Table 2).

**Table 1:** Baseline characteristics of all patients in the follow up cohort

**Number of patients**	**147**
Age, years (mean, sd*)	58 (12)
Female, N^o^ (%)	101 (69)
Weight, kg (mean, sd)	73 (14)
Disease duration, years (mean, sd)	11 (7)
RF positive, N^o^ (%)	117 (81)
Anti-CCP positive, N^o^ (%)	95 (69)
DAS28 at inclusion (mean, sd)	3.5 (1.3)
Interval duration at inclusion, weeks (median, IQR)	8 [4-8]
Previous DMARDs, weeks (median, IQR**)	3 [2,3]
Concomitant DMARD use, N^o^ (%)	111 (78)
Concomitant MTX use, N^o^ (%)	95 (67)
Previous anti-TNF-alpha therapy, N^o^ (%)	16 (11)
Duration of infliximab therapy, years (mean, sd)	2.5 (2.0)

* sd = standard deviation.

** IQR= interquartile range.

**Table 2:** Percentage of low and high infliximab serum trough levels and presence of anti-infliximab antibodies in patients with low and high DAS28 at the first study visit

	**No infliximab serum trough level**	Low* infliximab trough levels	**Intermediate infliximab trough levels**	High** infliximab trough levels	**Anti-infliximab antibodies**	**Total**
**DAS28**	% (95%CI)	% (95%CI)	% (95%CI)	% (95%CI)	% (95%CI)	N^o^ patients
<2.6	13 (2–23)	23 (10–35)	48 (32–63)	18 (6–29)	13 (2–23)	40
≤3.2	13 (4–20)	18 (9–28)	55 (43–67)	14 (5–22)	11 (3–19)	65
>3.2	29 (19–39)	18 (10–27)	32 (22–42)	21 (12–30)	29 (19–39)	82

* Low serum trough levels are defined as <1.0 mg/L.

** High serum trough levels are defined as >5.0 mg/L.

### Low infliximab serum trough levels in patients with long term low disease activity

Since at study enrolment a relatively high percentage of low infliximab serum trough level was found in patients with low disease activity, we examined whether the presence of low serum trough levels was persistent in patients with stable low disease activity. During follow-up of 1.5 years 68 patients complied with the criterion of having a stable DAS28 < 3.2 during at least 3 visits. Indeed we found that 34% (95% CI: 23-46%) of the patients in this subgroup had consecutively low trough levels, with 41% of them showing anti-infliximab antibodies. Mean infusion interval in these patients was 8.1 weeks (sd 2.05) and no interval changes occurred.

### Presence of anti-infliximab antibodies

Anti-infliximab antibodies were found in 18% of all 1320 visits (95%CI:16–20) and 49 of the 147 RA patients (33%, 95%CI:26–41) had anti-infliximab antibodies at least once during follow up. There was no significant association between MTX use and anti-infliximab antibodies as calculated by RR (0.75, 95%CI = 0.47-1.21, p = 0.25). In 22 out of 49 patients (45%) anti-infliximab antibodies were persistently found every visit. These patients had a median observation period of 58 weeks (IQR: 8–74) and a mean DAS28 of 3.2 (sd1.2). During follow up, 14 of the patients with anti-infliximab antibodies discontinued infliximab for the following reasons: ineffectiveness (n = 10, n = 2 also had an allergic reaction), allergic reaction (n = 1), malignant disease (n = 1), pregnancy wish (n = 1), other side effect (n = 1). In contrast 26 patients without anti-infliximab antibodies discontinued of whom 16 because of ineffectiveness.

There were 27 patients with intermittent anti-infliximab antibodies (median of 3 subsequent visits (IQR: 1–7)). The RR for the emerging of anti-infliximab antibodies after interval increase was 5.2 (95%CI 2.6-10.7, p < 0.0001). After interval decrease the RR for disappearing of anti-infliximab antibodies was 4.9 (95%CI 2.5-9.4, p < 0.0001). Interval changes occurred in 235 of the 1320 visits, of which 125 were interval increases. In 11 visits the interval increase was followed by anti-infliximab antibody emergence. Interval decreases occurred in 110 visits, with subsequent disappearance of anti-infliximab antibodies in 12 visits.

### Reliability of infliximab serum trough levels and anti-infliximab antibodies

**Figure 1:**
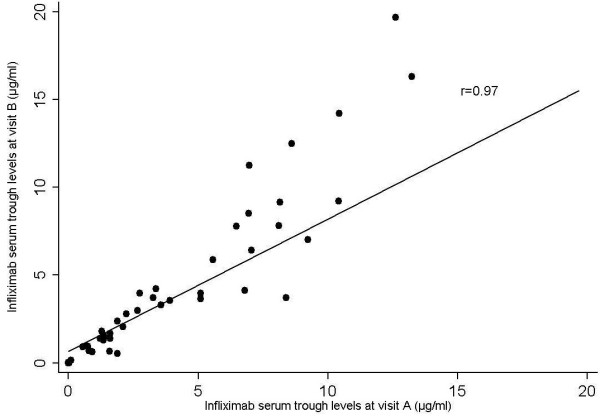
Serum trough levels measured at 2 consecutive visits in RA patients with stable DAS28 and infliximab dosing.

A small and non-significant mean difference in trough levels was found between two consecutive visits in patients with stable DAS28 and treatment (−0.26, p = 0.38), with relatively small LoA of −3.6 to 3.11. When considering serum trough levels in the relevant range of 0-5 mg/L, LoA were even smaller (−0.9 – 0.8), with a mean difference of −0.04. Agreement between serum trough levels analysed by spearman correlation was 0.97 (p = 0.00001, Figure 1). For anti-infliximab antibodies the correlation between subsequent visits was perfect with a kappa of 1.0 (standard error (SE) 0.14).

## Discussion

In our prospective observational longitudinal cohort study of RA patients treated with infliximab in the maintenance phase we encountered several interesting findings concerning the relation between disease activity, infliximab serum trough levels and anti-infliximab antibodies, and the influence of treatment interval changes.

First of all, low infliximab serum trough levels (with/without anti-infliximab antibodies) are prevalent in well-controlled RA patients treated with infliximab during the maintenance phase (about 1/3 of the patients). Since most articles on serum trough levels and anti-infliximab antibodies are describing the relation between low levels and non-response and high levels and good-response, this might seem a counterintuitive result. Nevertheless when examining these other studies one can also appreciate that a considerable proportion of patients with good response or low DAS28 has no measurable trough level of infliximab or have anti-infliximab antibodies (for example Pascual-Salcedo et al. also described that 24% of EULAR good-moderate responders to infliximab had anti-infliximab antibodies) [13]. Pondering on these findings it could be hypothesised that the low disease activity in these patients might not be attributed to infliximab. The initial response of infliximab in the induction phase of the treatment in these patients could be explained by a temporary need for infliximab, placebo response, regression to the mean or effects of co-medication.

There are some counterarguments against this line of reasoning. One could argue, for example, that the infliximab effect is (partially) determined by peak levels or time integrated area under the curve (AUC) rather than by minimal inhibitory concentration (MIC). This seems unlikely as subcutaneous anti-TNF agents demonstrate similar efficacy compared to intravenous anti-TNF without peak levels [18]. Also, it could be conceived that low infliximab serum levels and anti-infliximab antibodies just before the next infusion isn’t a good proxy for low serum levels or anti-infliximab antibodies during the majority of the treatment interval. However, Van den Bemt et al. demonstrated that anti-infliximab antibodies at the end of the interval is strongly associated with anti-infliximab antibodies or low/non-measurable serum levels in between infusions, indicating that low or no infliximab exposure was present during most of the treatment interval [19]. Nevertheless, whether RA patients with low infliximab serum trough levels can really de-escalate or stop treatment needs confirmation by means of an intervention study. Of note, high infliximab serum trough levels also occur relatively frequent. This may identify patients in whom the infliximab dose can be reduced whilst maintaining clinical efficacy.

A second finding was that reliability for consecutive measurements of serum trough levels and anti-infliximab antibodies of both infliximab levels and anti-infliximab antibody status was found to be very high. Reliable measurements are essential before considering measuring infliximab serum trough levels and anti-infliximab antibodies in daily clinical practice.

With regard to the anti-infliximab antibodies it should be noted that, although one-third of the patients had detectable antibodies during at least one visit, in only half of these patients they were found consistently every visit. Since the level of anti-infliximab antibodies is the resultant of antibody formation and (antigen-bound) antibody clearance, a change in infusion interval (thus a change in antigen challenge) might explain why in some patients anti-infliximab antibodies were only measurable temporarily. Indeed, after increasing the infusion interval anti-infliximab antibodies were found more frequently as can be concluded based on the RR. However, we have shown that, although anti-infliximab antibody status might change, most of these patients still have very low serum infliximab trough levels in between visits without antibodies. In addition our data failed to confirm the relationship between methotrexate use and reduced occurrence of anti-infliximab antibodies, which could well be due to a lack of statistical power [3].

Finally, our study confirmed earlier data that adequate disease control in daily clinical practice is often not reached, as demonstrated by less than half of the patients having a DAS28 below or equal to 3.2. Although the effect of tight control is well established [20,21] and a local protocol encouraged and facilitated physicians to change treatment if disease activity was inadequately controlled, still half of the patients didn’t reach the treatment target. This finding, that is concordant with findings in other biologic cohorts, underscores the need for better implementation strategies in daily clinical practice of tight control based treatment.

## Conclusions

In conclusion, our study demonstrated that measuring infliximab serum trough levels and anti-infliximab antibodies during the maintenance phase of infliximab treatment could be of value to optimise treatment. Low (and to some extent high) infliximab serum trough levels are prevalent in RA patients treated for a longer time with infliximab, also in patients with low disease activity. Since infliximab serum trough levels and anti-infliximab antibodies are reliable measurements, these might be used to identify patients in whom infliximab can be de-escalated or stopped.

## Abbreviations

AUC: Area under the curve; CI: Confidence interval; DAS28: Disease activity score of 28 joints; LoA: Limits of agreement; MIC: Minimal inhibitory concentration; MREC: Medical Research Ethics Committee; MTX: Methotrexate; RA: Rheumatoid arthritis; RR: Relative risk ratios; SD: Standard deviation; SE: Standard error.

## Competing interests

Piet van Riel has received grants from Pfizer, Abbott, BMS, Roche. None of the grants were connected in any way to this study and therefore we believe there are no conflicts of interest.

## Authors’ contributions

AM aided in the data acquisition, performed the statistical analysis and interpretation of the data and wrote the manuscript. BB was responsible for the conception and design of the study and data acquisition. He aided with the statistical analysis and interpretation of the data and critically revised the manuscript. GW and FH aided in the design of the study and revising the manuscript critically. GW also carried out the immunoassays. PR aided in the analysis and interpretation of the data. He also revised the manuscript critically. AB, together with BB, was responsible for the conception and design of the study. He aided the statistical analysis and interpretation and critically revised the manuscript several times. All authors read and approved the final manuscript.

## Pre-publication history

The pre-publication history for this paper can be accessed here:

http://www.biomedcentral.com/1471-2474/13/184/prepub
